# Relationship Between Right Ventricular Function and Body Composition in Adolescents and Young Adults

**DOI:** 10.3390/diagnostics15192487

**Published:** 2025-09-29

**Authors:** Karolina Angela Sieradzka Uchnar, Stefan Toth, Ingrid Schusterova, Dominik Pella, Silvia Gurbalova, Tibor Poruban

**Affiliations:** 1 East Slovak Institute of Cardiovascular Diseases and School of Medicine, Pavol Jozef Safarik University, Ondavska 8, 040 01 Kosice, Slovakia; sieradzka.ina@gmail.com (K.A.S.U.); ischusterova@gmail.com (I.S.); dominik.pella@gmail.com (D.P.); siska.buzova@gmail.com (S.G.); 2 Gerontology and Geriatrics Clinic, Pavol Jozef Safarik University, Trieda SNP 1, 040 01 Kosice, Slovakia; stefan.toth@me.sk

**Keywords:** body composition, echocardiography, obesity, overweight, right ventricle

## Abstract

**Objective:** This study sought to examine the relationships between right ventricular (RV) parameters and function, and body composition in adolescents and young adult individuals with overweight and obesity. We hypothesized that abnormal body composition is linked to RV dysfunction and subclinical changes in the ventricle. **Methods:** The study prospectively included 80 young adult individuals, with 55 being overweight or obese and 25 having a normal body weight. We examined differences in RV echocardiographic parameters between these groups and their relationship with body composition parameters. **Results:** Adolescents and young adults with overweight or obesity had greater RV pressure load, and larger RV diameter. Significant differences in RV size and strain were noted between groups. Across the cohort, lean body mass positively correlated with RV strain, while fat mass and total serum protein negatively correlated with RV strain (both *p* < 0.01 or lower). **Conclusions:** This study found that RV function and body composition are often linked and improving body composition could prevent RV dysfunction, while addressing wasting might enhance RV function. Overweight or obese young adults show decreased RV strain in the absolute value compared to those with normal body weight.

## 1. Introduction

Obesity is a major risk factor for cardiovascular disease and early mortality, driving atherosclerosis, hypertension, diabetes, and heart failure. The concept of obesity cardiomyopathy highlights the direct effects of adipose tissue–mediated metabolic, hemodynamic, and inflammatory changes on cardiac structure and function, independent of comorbidities [1,2,3]. These mechanisms include increased blood volume, neurohormonal activation, myocardial fat infiltration, and systemic inflammatory signaling, leading to both left and right ventricular remodeling [4,5].

Although obesity’s link to left ventricular dysfunction is well known, advanced imaging shows it also adversely affects the right ventricle by increasing mass and volumes and reducing ejection fraction, independent of left ventricular changes [6]. Severe obesity further impairs RV energetics and pulmonary hemodynamics, whereas substantial weight loss can partially reverse these changes [7]. These findings highlight the need for biventricular assessment in obese patients and show that weight trajectory strongly predicts outcomes, with guidelines recommending individualized weight management [8].

Body composition is critical, as visceral fat and muscle loss worsen loading conditions, pulmonary pressures, and metabolism, leading to RV dysfunction [9]. Visceral fat promotes vascular dysfunction, while sarcopenia lowers reserve; studies confirm impaired RV function and exercise capacity in obesity [10].

Direct invasive hemodynamic studies in obese patients with unexplained exercise intolerance have demonstrated that both obese individuals with and without exercise-induced pulmonary venous hypertension exhibit impaired RV-arterial coupling during exercise, compared to controls. This uncoupling is characterized by a failure of RV contractile reserve to match the increased afterload during exercise, resulting in reduced cardiac output and impaired exercise capacity [11,12,13].

Based on these insights, the hypothesis was that adverse body composition is associated with subclinical changes in RV geometry and function even in younger individuals without comorbidities. To reduce the risk of statistical multiplicity, analyses were limited to predefined, prognostically relevant RV parameters [14].

## 2. Materials and Methods

### 2.1. Study Population

**Figure 1:**
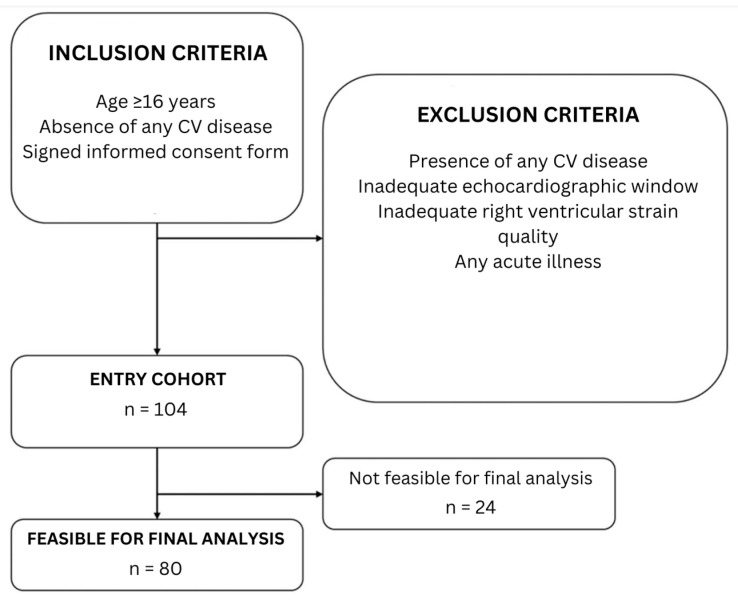
A flowchart of patient enrollment is shown (authors).

The study is a prospective single-center cohort study conducted at the Department of Functional Diagnostics of the academic tertiary referral center of the East Slovak Institute of Cardiovascular Diseases and School of Medicine, Pavol Jozef Safarik University, Kosice, Slovakia. Between June 2022 and September 2023, a total of 104 consecutive patients were enrolled. The main inclusion criteria were age above 16 years, absence of any diagnosed cardiovascular disease, and/or participation in other studies. Exclusion criteria included the presence of any diagnosed cardiovascular disease, including arterial hypertension, diabetes mellitus, or dyslipidemia, inadequate echocardiographic window and/or RV strain quality (as a result of the aforementioned factor). Final study cohort included 80 young adult individuals (47 men and 33 women) aged 16 to 24 years, who were divided into two groups based on body mass index (BMI) values—those with normal weight (BMI = 18.5–24.9 kg/m^2^) and those overweight or obese (BMI = ≥25 kg/m^2^) (Figure 1).

### 2.2. Echocardiographic Assessment

Each patient underwent a comprehensive transthoracic echocardiographic examination using the Philips EPIQ CVx device (Philips, Eindhoven, The Netherlands), which was performed by the same cardiologist. All parameters were recorded in accordance with the current recommendations of the European Association of Cardiovascular Imaging or the American Society of Echocardiography using standard modalities such as 2D, M-mode, pulse, tissue, and continuous wave Doppler [15,16,17,18].

The following echocardiographic parameters of the RV were monitored: the amplitude of lateral tricuspid annular motion (TAPSE) and its maximum velocity during systole (S’), systolic pressure in the pulmonary artery (PASP), basal and mid-ventricular diameter of the RV (RV_BD_, RV_MD_), fractional area change (FAC), two-dimensional longitudinal strain of the free wall of the RV (2D RV_FWLS_), and two-dimensional global longitudinal strain of the RV (2D RV_4CLS_). The tricuspid regurgitation (TR) was assessed using a semi-quantitative, integrative method based on key indicators included jet area, vena contracta width, Doppler jet density, hepatic vein flow, trans-tricuspid inflow pattern, annular diameter, and RV/RA size. These parameters were analyzed for concordance [19].

**Figure 2:**
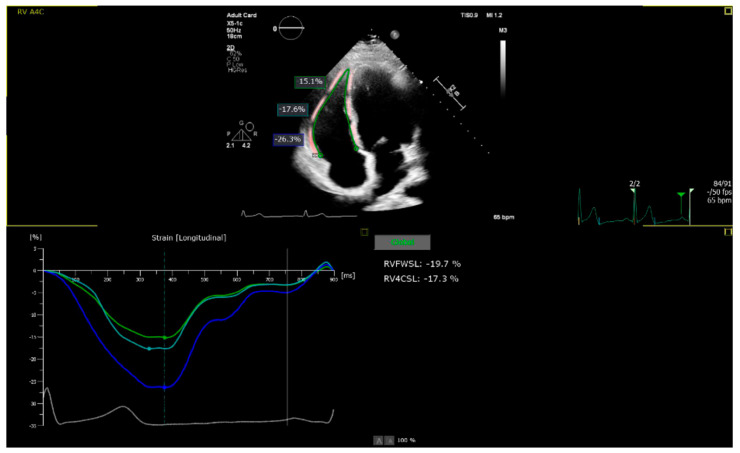
Apical right ventricular—focused four-chamber view obtained by two-dimensional echocardiographic acquisitions and automatic analysis of the right ventricular strain using two-dimensional speckle transthoracic echocardiography (authors).

All images were acquired in the focused (on the RV) apical four-chamber view and exported digitally (Figure 2). Subsequently, the parameters 2D RV_FWLS_ and 2D RV_4CLS_ were analyzed using AutoStrain RV 5.0 (Philips, Eindhoven, The Netherlands), which automatically generated RV strain curves and evaluated the 2D RV_FWLS_ parameter as the average value of these curves at the basal, mid-ventricular, and apical segments. For each patient, the analysis was performed across different cardiac cycles from the same cine loops, when accessible, and the results were averaged. The right ventricular (RV) strain was measured as endomyocardial strain using a six-segment model and is referred to in the text as an absolute value. A frame rate of clips used for analysis, including global longitudinal strain, was 60 frames per second, in accordance with recent guidelines [20].

### 2.3. Laboratory Parameters

The assessment of laboratory parameters included hepatic parameters (alanine transaminase, ALT; aspartate transaminase, AST; gamma-glutamyl transpeptidase, GGT), lipidogram (total cholesterol, CHOL; high-density lipoprotein cholesterol, HDL; low-density lipoprotein cholesterol, LDL; triglycerides, TAG), albumin, uric acid, total serum protein, C-peptide, and insulin. Blood sampling was performed, and laboratory parameters immediately determined at the East Slovak Institute of Cardiovascular Diseases, Kosice, Slovakia.

### 2.4. Body Composition Scale

Each patient, with the cooperation of the at Children’s Faculty Hospital in Kosice, Slovakia, underwent body composition evaluation. Using advanced technology to guarantee accuracy and reproducibility, body composition analysis was performed using an InBody 520 analyzer. InBody^®^ works on the principle of bioelectrical impedance analysis—a technique used to assess body composition, including muscle mass, body fat, and total body water (TBW). It works by sending alternating low- and high-frequency electrical currents through the body via electrodes, leveraging the water content in tissues to measure impedance. This impedance value helps calculate TBW, which in turn is used to estimate fat-free mass (comprising muscles, bones, and other lean tissue) and body fat. The ability to differentiate between extracellular water (ECW) and intracellular water (ICW) is essential for identifying conditions such as inflammation or edema. Many BIA devices operate at a single frequency (typically 50 kHz), which cannot fully penetrate cell membranes, limiting their ability to measure TBW accurately. This limitation can result in undetected health risks, especially in individuals with abnormal ECW levels. In contrast, InBody^®^ devices utilize multiple frequencies, enabling precise measurement of ECW, ICW, and TBW. This multi-frequency approach improves the accuracy of body composition analysis and fluid status evaluation. Unlike traditional BIA devices, InBody^®^ measurements are not influenced by empirical assumptions related to age, sex, ethnicity, or body shape, ensuring highly accurate and reliable results validated against gold-standard methods. Medical practitioners widely use InBody^®^ for comprehensive assessments of body composition and hydration status [21].

### 2.5. Statistical Analysis

The data are presented using descriptive statistics. Continuous variables are reported as mean ± standard deviation or median with interquartile range, depending on distribution. Categorical variables are shown as percentages. Normality was assessed using Kolmogorov–Smirnov and Shapiro–Wilk tests. Welch’s *t*-test compared continuous variables, while Chi-square or Fisher’s Exact test compared categorical variables. Analyses were conducted in SPSS Statistics 20 (IBM, Armonk, NY, USA), with statistical significance defined as a two-tailed *p*-value < 0.05. To prevent false positive results, Bonferroni adjustment was also used in the final analysis.

## 3. Results

The study included 80 young adults, with 25 having normal weight (BMI 18.5–24.9 kg/m^2^), 28 classified as overweight (BMI 25.0–29.9 kg/m^2^), and 27 classified as obese (BMI ≥ 30 kg/m^2^). The average age was comparable across groups (normal weight: 20.4 ± 2.96 years; overweight: 19.6 ± 2.7 years; obese: 19.4 ± 2.9 years). Mean BMI was 21.5 ± 1.91 kg/m^2^ in the normal weight group, 27.2 ± 1.3 kg/m^2^ in the overweight group, and 34.5 ± 3.1 kg/m^2^ in the obese group. Body composition analysis revealed a progressive increase in fat mass from normal weight (9.8 ± 5.4 kg) to overweight (19.7 ± 7.6 kg) to obese individuals (31.8 ± 9.9 kg), while lean body mass also increased stepwise across these categories (52.0 ± 10.1, 60.4 ± 10.7, and 65.8 ± 11.2 kg, respectively) (Table 1).

### 3.1. Echocardiographic Findings

The left ventricular ejection fraction (LVEF) was preserved across all groups (normal weight: 58.4 ± 4.3%, overweight: 57.1 ± 3.8%, obese: 56.4 ± 3.9%). Pulmonary artery systolic pressure (PASP) and RV diameters showed a stepwise increase with higher BMI (PASP: 22.7 ± 4.7 mmHg, 25.1 ± 5.1 mmHg, 27.9 ± 5.8 mmHg; RV basal diameter: 31.9 ± 3.9 mm, 34.0 ± 3.5 mm, 36.7 ± 3.2 mm for normal weight, overweight, and obese, respectively; *p*-trend < 0.001).

**Figure 3:**
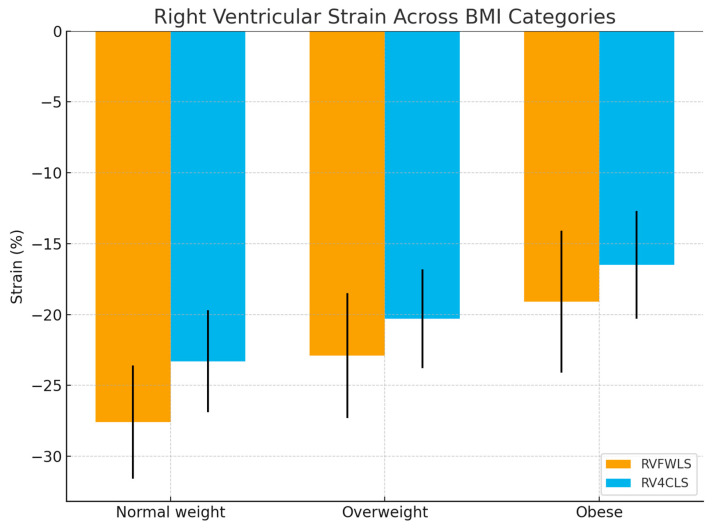
Graded decline in 2D RV_FWLS_ and RV_4CLS_ across study groups (authors). 2D RV_FWLS_—two-dimensional right ventricular free-wall longitudinal strain, 2D RV_4CLS_—two-dimensional right ventricular global longitudinal strain.

RV functional parameters also showed a graded impairment across BMI categories. RV free wall longitudinal strain (RV_FWLS_) declined from −27.6 ± 4.0% in normal weight to −22.9 ± 4.4% in overweight and −19.1 ± 5.0% in obese individuals. Similarly, RV four-chamber longitudinal strain (RV_4CLS_) decreased stepwise (−23.3 ± 3.6%, −20.3 ± 3.5%, and −16.5 ± 3.8%, respectively; both *p*-trend < 0.001). These graded differences are illustrated in Figure 2 and Figure 3.

### 3.2. Correlations with Body Composition

**Figure 4:**
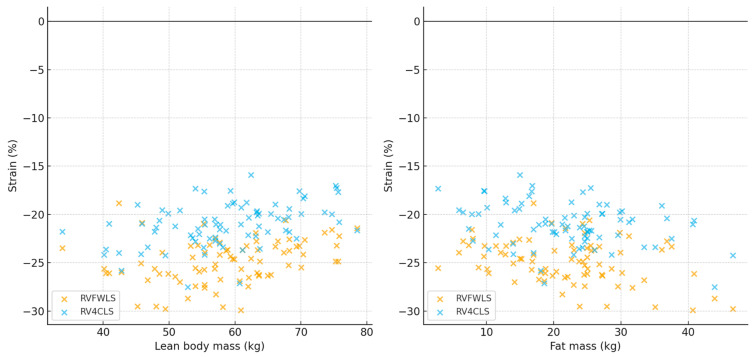
Correlations of Lean Body mass (LBM) and Fat mass (FM) with RV strain (authors).

In the total cohort, lean body mass correlated positively with RV strain (RV_FWLS_ r = 0.29, *p* < 0.01; RV_4CLS_ r = 0.31, *p* < 0.001), whereas fat mass correlated negatively (RV_FWLS_ r = −0.17, *p* < 0.05; RV_4CLS_ r = −0.27, *p* < 0.001). Total protein levels were also inversely associated with RV strain (RV_FWLS_ r = −0.18, *p* < 0.01; RV_4CLS_ r = −0.22, *p* < 0.05). These relationships are shown in Figure 4, and Table 2, Table 3 and Table 4, respectively.

In our analysis, RV strain values were expressed as absolute values (i.e., positive numbers), as is standard in most speckle-tracking software packages. Therefore, a lower strain value corresponds to impaired myocardial deformation. This convention allows for easier clinical interpretation but requires clarification when presenting correlations, since negative r values then indicate that higher fat mass or protein levels are associated with less favorable (i.e., lower) strain magnitudes.

### 3.3. Trend Analysis

The progressive changes in RV size and strain across normal weight, overweight, and obese groups support a dose–response relationship between abnormal body composition and RV remodeling, while correlation analyses confirm that both lean and fat mass independently contribute to RV function.

Although TAPSE and S′ values were similar across groups, RV strain parameters demonstrated significant differences. This discrepancy is consistent with the fact that TAPSE and S′ reflect only basal RV longitudinal motion and are highly load-dependent, whereas strain imaging integrates deformation across the entire RV free wall or chamber and is more sensitive to early, subclinical dysfunction. Thus, strain may detect obesity-related myocardial changes before they are apparent in conventional metrics.

## 4. Discussion

Recent evidence demonstrates that body composition independently influences RV structure and function in adolescents and young adults, with both RV dysfunction and abnormal body composition affecting cardiac mechanics [22,23,24,25]. Assessment of RV mechanics using advanced echocardiographic parameters, such as longitudinal strain, is increasingly recognized as a sensitive method for detecting early, subclinical myocardial changes in overweight and obese youth—a population in which conventional measures like ejection fraction may fail to detect dysfunction [26,27,28,29].

In healthy, non-obese individuals, lean body mass correlates with RV size (base and mid-diameter), which may reflect early remodeling rather than improved function. Increased fat mass is associated with reduced RV strain at the base, while in obese adolescents, lean body mass predicts strain in the apical free wall of the RV, demonstrating the complex interplay between body compartments and RV mechanics [26].

Although adult studies robustly link obesity to RV remodeling, pediatric and young adult data have been inconsistent [30]. Recent imaging and Mendelian randomization studies indicate that increased BMI and altered body composition in youth are associated with larger RV volumes, increased RV mass, and impaired RV strain, even without overt clinical disease [31,32]. Strain imaging remains superior to ejection fraction for detecting early RV dysfunction, with most studies, including the present findings, showing a reduction in RV strain of approximately 20–25% [26,27,28].

Mechanistically, excess adiposity induces metabolic disturbances—including insulin resistance, chronic low-grade inflammation, and altered adipokine signaling—that promote myocardial hypertrophy, fibrosis, and chamber dilation [9,25,30]. These changes affect both ventricles, but the RV is particularly susceptible due to its thin wall and complex geometry [32,33]. Obesity-related metabolic derangements, such as elevated proinflammatory cytokines and lipotoxicity, directly impair RV myocyte function. Indirectly, obesity increases RV preload and afterload via expanded blood volume, higher pulmonary pressures, and sleep-disordered breathing [3,30,34,35]. Mendelian randomization and imaging studies confirm causal associations between higher BMI, visceral adiposity, increased RV mass, larger RV volumes, and impaired strain in youth without overt cardiovascular disease [9,20,28].

Echocardiography remains the most widely used modality for RV assessment in this population, but its accuracy is limited by poor acoustic windows and suboptimal image quality in individuals with obesity [35]. Cardiac magnetic resonance imaging (CMR) offers superior spatial resolution and reproducibility for RV structure and function, and recent studies advocate for its use as the gold standard, especially in research settings and for detailed phenotyping [20,21,25,28]. Future investigations should systematically compare echocardiography and CMR to clarify the impact of imaging modality on detection and characterization of subclinical RV changes.

The prognostic significance of RV dysfunction in the context of obesity is increasingly recognized, with recent data showing that RV dysfunction confers a higher risk of mortality and heart failure hospitalization, particularly in those with elevated BMI [36]. However, the role of the RV in early risk stratification and prediction of subclinical myocardial changes in youth remains underexplored, and larger, longitudinal studies are needed to elucidate these relationships and inform clinical management [23,25,28,36].

## 5. Limitations

The principal limitation of this study lies in its specialized focus on subclinical alterations in RV function, particularly in the absence of standardized protocols for three-dimensional (3D) RV analysis. At present, there is no universally accepted methodology for quantifying these subtle changes, necessitating reliance on published meta-analyses and observational data rather than consensus recommendations or guidelines.

Another constraint is the requirement for high-quality imaging in all study participants. Acquisition of adequate transthoracic echocardiographic images can be challenging, especially in individuals with increased BMI, which may result in exclusion of certain patients and introduce selection bias. Only those meeting strict imaging criteria were included, potentially limiting the representativeness of the cohort.

Additional limitations stem from the single-center, retrospective design, which in-herently restricts the sample size and diversity of the study population. The relatively small number of participants is a consequence of both the rarity of the diagnosis and stringent inclusion and exclusion criteria. As this was a non-randomized study conducted in a tertiary care setting, baseline characteristics such as functional status were not evenly distributed, which may influence the generalizability of the findings to broader patient’s populations. Finally, absence of the reference absorptiometric method is another limitation that needs to be mentioned.

## 6. Conclusions

This study demonstrated that RV function and body composition are associated, potentially having a mechanistic connection, and together they impact the health status of young patients. It seems that improving body composition could prevent RV dysfunction, while reversing a wasting state might enhance RV function [37]. Based on the results of our study, it appears that young adults with overweight or obesity exhibit early signs of RV dysfunction compared to their peers with a normal body habitus. Worsening values of RV_FWLS_ and RV_4CLS_ suggest subclinical myocardial dysfunction, despite normal values of standard parameters (TAPSE, S’). To confirm these findings, more detailed research is needed, including the assessment of body fat percentage and its distribution in the body, which would help better understand the relationship between obesity and impaired myocardial function.

## Figures and Tables

**Table 1:** Characteristics of the participants in the study.

	Overweight or Obesity N = 55	Normal Weight N = 25	*r*	*p*
**Demographics**				
Female, *n* (%)	23 (42)	10 (40)		0.081
Age, years	19.5 ± 2.8	20.4 ± 2.96		0.816
Weight (kg)	77.1 ± 3.9	71.8 ± 2.7		**0.005**
Height (cm)	173.2 ± 4.7	175.4 ± 4.5		**0.005**
BMI (kg/m^2^)	31.4 ± 4.0	21.5 ± 2.0		***p* < 0.0001**
Caucasian ethnicity (%)	55 (100)	25 (100)		
**Clinical characteristics**				
SBP, mmHg	122 ± 21	121 ± 19		**0.008**
DBP, mmHg	75 ± 12	77 ± 13		**0.007**
24 h SBP, mmHg	109 ± 11	111 ± 13		**0.005**
24 h DBP, mmHg	66 ± 9	67 ± 9		0.076
Heart rate, bpm	64 ± 10	65 ± 11		0.214
24 h heart rate, bpm	65 ± 10	67 ± 11		0.459
Smoker, *n* (%)	9 (16)	4 (16)		0.573
**Laboratory parameters**				
AST, μkat/L	0.47 ± 0.21	0.41 ± 0.13	−0.18	0.249
ALT, μkat/L	0.46 ± 0.31	0.31 ± 0.16	−0.16	0.061
GGT, μkat/L	0.39 ± 0.24	0.26 ± 0.07	−0.05	0.819
CHOL, mmol/L	4.33 ± 0.64	4.24 ± 0.74	−0.19	0.312
TAG, mmol/L	1.15 ± 0.54	0.79 ± 0.40	−0.09	0.624
LDL, mmol/L	2.84 ± 0.72	2.54 ± 0.62	−0.22	0.238
HDL, mmol/L	1.26 ± 0.42	1.57 ± 0.41	−0.04	0.796
Albumin, g/L	45.62 ± 2.23	45.65 ± 2.55	0.09	0.085
Uric acid, mmol/L	325.89 ± 80.26	269.83 ± 82.70	0.08	0.552
Total serum protein, kg	72.46 ± 2.97	73.85 ± 3.73	0.10	0.074
C-peptide, nmol/L	1242.61 ± 619.82	712.1 ± 385.2	0.13	0.306
Insulin, mmol/L	13.37 ± 6.57	10.01 ± 6.90	0.05	0.495
**Body composition**				
Total water, L	45.53 ± 8.64	38.21 ± 7.45	0.24	0.083
Total minerals, kg	4.03 ± 0.83	3.47 ± 0.60	0.29	0.057
Fat mass, kg	25.46 ± 13.39	9.83 ± 5.36	−0.41	**0.035**
Lean body mass, kg	62.84 ± 11.53	52.03 ± 10.11	0.59	**0.009**
**Echocardiography**				
RV_BD_ (mm)	35.4 ± 3.6	31.9 ± 3.9	0.67	***p* < 0.05**
RV_MD_ (mm)	27.8 ± 3.9	25.7 ± 3.9	0.12	***p* < 0.04**
FAC (%)	44.7 ± 5.0	46.4 ± 5.6	0.15	ns
2D RV_FWLS_ (%)	20.8 ± 5.2	27.6 ± 4.0	0.32	***p* < 0.001**
2D RV_4CLS_ (%)	18.2 ± 3.8	23.3 ± 3.6	0.29	***p* < 0.001**
LVEF, %	56.70 ± 3.90	58.40 ± 4.25	−0.24	***p* <0.01**
S’(cm/s)	13.1 ± 1.6	14.1 ± 1.64	−0.09	***p* < 0.01**
TAPSE, mm	22.93 ± 2.5	23.1 ± 2.5	−0.16	ns
PASP, mmHg	26.57 ± 5.7	22.7 ± 4.7	0.21	***p* < 0.01**

2D RVFWLS—two-dimensional right ventricular free-wall longitudinal strain, 2D RV4CLS—two-dimensional right ventricular global longitudinal strain, ALT—alanine transaminase, AST—aspartate transaminase, BMI—body mass index, CHOL—cholesterol, DBP—diastolic blood pressure, FAC—fraction area change, GGT—gamma-glutamyl transpeptidase, HDL—high-density lipoprotein cholesterol, LDL—low-density lipoprotein cholesterol, PASP—pulmonary arterial systolic pressure; r—Pearson’s correlation coefficient; RVBD—right ventricle base diameter, RVMD—right ventricle mid diameter, S’—peak systolic velocity of the tricuspid annulus, SBP—systolic blood pressure, TAG—triglycerides, TAPSE—tricuspid annular plane systolic excursion; ns—not significant.

**Table 2:** Correlations between right ventricular parameters and body composition—both groups.

Echocardiographic Parameters	Body Composition Parameters									
	TP		TW		MIN		FM		LBM	
	r	*p*	r	*p*	r	*p*	r	*p*	r	*p*
**S’ (cm/s)**	−0.10	**<0.01**	−0.09	ns	−0.06	ns	−0.02	**<0.01**	−0.11	**<0.01**
**TAPSE, mm**	−0.17	ns	−0.15	ns	−0.01	ns	−0.02	ns	−0.11	ns
**PASP, mmHg**	0.19	**<0.01**	0.12	ns	0.09	ns	0.17	**<0.01**	0.23	**<0.01**
**RV_BD_ (mm)**	0.05	**<0.01**	−0.04	ns	0.12	ns	0.10	**<0.001**	0.05	**<0.01**
**RV_MD_ (mm)**	0.12	**<0.05**	0.09	ns	0.13	ns	0.32	**<0.04**	0.14	**<0.04**
**FAC (%)**	0.24	ns	0.23	ns	0.15	ns	0.30	ns	0.25	ns
**2D RV_FWLS_ (%)**	−0.25	**<0.01**	0.28	ns	0.32	ns	−0.22	**<0.001**	0.29	**<0.01**
**2D RV_4CLS_ (%)**	−0.24	**<0.01**	0.26	ns	0.29	ns	−0.29	**<0.001**	0.29	**<0.001**

2D RV_FWLS_—two-dimensional right ventricular free-wall longitudinal strain, 2D RV_4CLS_—two-dimensional right ventricular global longitudinal strain, FAC—fraction area change, FM—fat mass, LBM—lean body mass, MIN—minerals, ns—not significant, PASP—pulmonary arterial systolic pressure; r—Pearson’s correlation coefficient; RV_BD_—right ventricle base diameter, RV_MD_—right ventricle mid diameter, S’—peak systolic velocity of the tricuspid annulus, TAPSE—tricuspid annular plane systolic excursion, TP—total protein, TW—total water.

**Table 3:** Correlations between right ventricular parameters and body composition—overweight and obese group.

Echocardiographic Parameters	Body Composition Parameters									
	TP		TW		MIN		FM		LBM	
	r	*p*	r	*p*	r	*p*	r	*p*	r	*p*
**S’ (cm/s)**	−0.12	**<0.01**	−0.08	ns	−0.05	ns	−0.03	**<0.01**	−0.09	**<0.01**
**TAPSE, mm**	−0.14	ns	−0.13	ns	−0.02	ns	−0.01	ns	−0.12	ns
**PASP, mmHg**	0.21	**<0.01**	0.11	ns	0.07	ns	0.18	**<0.01**	0.22	**<0.01**
**RV_BD_ (mm)**	0.06	**<0.01**	−0.03	ns	0.10	ns	0.11	**<0.05**	0.06	**<0.01**
**RV_MD_ (mm)**	0.14	**<0.05**	0.10	ns	0.09	ns	0.33	**<0.05**	0.15	**<0.04**
**FAC (%)**	0.21	ns	0.20	ns	0.18	ns	0.28	ns	0.23	ns
**2D RV_FWLS_ (%)**	−0.28	**<0.01**	0.27	ns	0.30	ns	−0.17	**<0.001**	0.29	**<0.01**
**2D RV_4CLS_ (%)**	−0.22	**<0.05**	0.21	ns	0.28	ns	−0.27	**<0.001**	0.31	**<0.001**

2D RV_FWLS_—two-dimensional right ventricular free-wall longitudinal strain, 2D RV_4CLS_—two-dimensional right ventricular global longitudinal strain, FAC—fraction area change, FM—fat mass, LBM—lean body mass, MIN—minerals, ns—not significant, PASP—pulmonary arterial systolic pressure; r—Pearson’s correlation coefficient; RV_BD_—right ventricle base diameter, RV_MD_—right ventricle mid diameter, S’—peak systolic velocity of the tricuspid annulus, TAPSE—tricuspid annular plane systolic excursion, TP—total protein, TW—total water.

**Table 4:** Correlations between right ventricular parameters and body composition—normal weight group.

Echocardiographic Parameters	Body Composition Parameters									
	TP		TW		MIN		FM		LBM	
	r	*p*	r	*p*	r	*p*	r	*p*	r	*p*
**S’ (cm/s)**	−0.11	**<0.01**	−0.07	ns	−0.08	ns	−0.04	**<0.01**	−0.10	**<0.001**
**TAPSE, mm**	−0.16	ns	−0.12	ns	−0.03	ns	−0.01	ns	−0.09	ns
**PASP, mmHg**	0.18	**<0.05**	0.13	ns	0.10	ns	0.16	**<0.01**	0.21	**<0.01**
**RV_BD_ (mm)**	0.07	**<0.01**	−0.02	ns	0.09	ns	0.12	**<0.001**	0.07	**<0.01**
**RV_MD_ (mm)**	0.14	**<0.05**	0.08	ns	0.12	ns	0.30	**<0.04**	0.13	**<0.04**
**FAC (%)**	0.23	ns	0.22	ns	0.14	ns	0.27	ns	0.26	ns
**2D RV_FWLS_ (%)**	−0.27	**<0.01**	0.29	ns	0.30	ns	−0.23	**<0.01**	0.28	**<0.01**
**2D RV_4CLS_ (%)**	−0.20	**<0.001**	0.23	ns	0.25	ns	−0.30	**<0.001**	0.28	**<0.001**

2D RV_FWLS_—two-dimensional right ventricular free-wall longitudinal strain, 2D RV_4CLS_—two-dimensional right ventricular global longitudinal strain, FAC—fraction area change, FM—fat mass, LBM—lean body mass, MIN—minerals, ns—not significant, PASP—pulmonary arterial systolic pressure; r—Pearson’s correlation coefficient; RV_BD_—right ventricle base diameter, RV_MD_—right ventricle mid diameter, S’—peak systolic velocity of the tricuspid annulus, TAPSE—tricuspid annular plane systolic excursion, TP—total protein, TW—total water.

## Data Availability

The raw data supporting the conclusions of this article will be made available by the authors on request.

## Abbreviations

2D RV_4CLS_	Two-dimensional global longitudinal strain of the right ventricle
2D RV_FWLS_	Two-dimensional longitudinal strain of the free wall of the right ventricle
3D	Three-dimensional
BMI	Body mass index
CMR	Cardiac Magnetic Resonance
CVD	Cardiovascular diseases
ECW	Extracellular water
EF	Ejection fraction
FAC	Change in fractional area change
HF	Heart failure
ICW	Intracellular water
LV	Left ventricle
PASP	Systolic pressure in the pulmonary artery
RV	Right ventricle
RV_BD_, RV_MD_	Basal and mid-ventricular diameter of the RV
S’	Maximum velocity of lateral tricuspid annular motion during systole
TAPSE	Amplitude of lateral tricuspid annular motion
TBW	Total Body Water
TR	Tricuspid regurgitation
